# Current status of radioligand therapy and positron-emission tomography with prostate-specific membrane antigen

**DOI:** 10.1007/s12149-020-01549-5

**Published:** 2020-11-11

**Authors:** Masayuki Inubushi, Hiroyuki Miura, Ichiei Kuji, Kimiteru Ito, Ryogo Minamimoto

**Affiliations:** 1 Division of Nuclear Medicine, Department of Radiology, Kawasaki Medical School, 577 Matsushima, Kurashiki, Okayama 701-0192 Japan; 2 Department of Radiology, Hirosaki University School of Medicine, 5 Zaifu-cho, Hirosaki, Aomori 036-8562 Japan; 3 Department of Nuclear Medicine, Saitama Medical University International Medical Center, 1397-1 Yamane, Hidaka, Saitama 350-1298 Japan; 4 Department of Diagnostic Radiology, National Cancer Center Hospital, 5-1-1 Tsukiji, Chuo-ku, Tokyo 104-0045 Japan; 5 Division of Nuclear Medicine, Department of Radiology, National Center for Global Health and Medicine, 1-21-1, Toyama, Shinjyuku-ku, Tokyo 162-8655 Japan

**Keywords:** Prostate-specific membrane antigen (PSMA), Radioligand therapy (RLT), Positron-emission tomography (PET), Theranostics, Radionuclides

## Abstract

Prostate-specific membrane antigen (PSMA) is a transmembrane glycoprotein highly expressed by prostate cancer cells. PSMA-based radioligand therapy (RLT) emerged as a promising therapeutic option for prostate cancer in the early 2000s, and has been clinically validated with great enthusiasm during these past two decades. Last year, the European Association of Nuclear Medicine (EANM) published the procedure guidelines for the safe clinical practice of Lutetium-177 (^177^Lu)-labelled PSMA RLT. In addition, PSMA RLT with alpha-ray-emitting radioisotopes has been also developed recently. Following the clinical use of ^177^Lu-PSMA RLT, PSMA-targeted positron-emission tomography (PET) with Gallium-68 (^68^Ga) has been performed inevitably for “theranostics” for the last decade; prostate cancer is going to be treated with PSMA-RLT based on the diagnosis by PSMA-PET. Furthermore, the diagnostic usefulness of ^68^Ga-PSMA PET has been documented in various diseases beyond prostate cancer more recently. Regrettably, Japan is behind European countries and the United States in this field, and has just made a belated start of their clinical trials. In this review article, we briefly overviewed the current status of PSMA RLT and PSMA PET. We hope that this topic will be a particular focus of attention for most ANM readers in Japan, and that our efforts will help to facilitate the early approval of PSMA RLT and PSMA PET by the Japanese government even if only slightly.

## Introduction

Prostate-specific membrane antigen (PSMA) is a transmembrane glycoprotein highly expressed by prostate cancer cells. PSMA-based radioligand therapy (RLT) emerged as a promising therapeutic option for prostate cancer in the early 2000s [1], and has been clinically validated with great enthusiasm during these past these two decades. Last year, the European Association of Nuclear Medicine (EANM) published the procedure guidelines for the safe clinical practice of Lutetium-177 (^177^Lu)-labelled PSMA RLT [2]. In addition, PSMA RLT with alpha-ray-emitting radioisotopes has also been developed recently.

Following the clinical use of ^177^Lu-PSMA RLT, PSMA-targeted positron-emission tomography (PET) with Gallium-68 (^68^ Ga) has been performed inevitably for “theranostics” for the last decade [3]; prostate cancer is going to be treated with PSMA RLT based on the diagnosis by PSMA PET. Dr. Jadvar, one of pioneers in the field of PSMA RLT, once said: “Theranostics is currently experiencing a renaissance since the early days of radioiodine use in thyroid diseases” [4]. Furthermore, the diagnostic usefulness of ^68^Ga-PSMA PET has been clarified in various diseases beyond prostate cancer more recently.

Regrettably, Japan is behind European countries and the United States in this field, and has just made a belated start of their clinical trials. In this review article, we briefly overview the current status of PSMA RLT and PSMA PET.

## Safety of ^177^Lu-PSMA radioligand therapy: EANM Procedure Guidelines and literature review

Last year, the EANM published procedure guidelines for ^177^Lu-PSMA RLT to assist nuclear medicine specialists in delivering PSMA-RLT as an “unproven intervention in clinical practice”, in accordance with the best currently available knowledge [2]. Presented in these safety guidelines are the results of recent phase II trials in which the most common toxic effects possibly related to ^177^Lu-PSMA-617 were grade 3 lymphocytopenia in 37% of patients, grade 3 anemia in 13%, and grade 3 or 4 thrombocytopenia in 13%. In addition, grade 1 dry mouth was reported in 87% of patients, grade 1 or 2 transient nausea in 50%, and grade 1 or 2 fatigue in 50%. The authors considered that these data indicated a favorable safety profile for ^177^Lu-PSMA RLT [2].

Banerjee et al. synthesized 14 new PSMA-targeted, ^177^Lu-labeled radioligands (^177^Lu-L1–^177^Lu-L14) [5] and investigated their pharmacokinetics, capacity to kill cells in vitro, and tumor control in vivo. In addition, they evaluated the cell uptake, internalization, biodistribution, efficacy, toxicity, tumor regression, and survival rate of these radioligands in mice. They optimized aspects of the radiosynthesis and assessed the short- and long-term toxicity of ^177^Lu-L1. Their self-blocking study showed that ^177^Lu-L1, which already demonstrates relatively low renal uptake as a type-II agent, caused no significant change in tumor uptake but yielded a ten-fold kidney blockade. They concluded that the scaffold on which ^177^Lu-L1 is based may also provide guidance for the corresponding alpha-ray emitters, which although potentially more efficacious, have the disadvantage of more severe adverse reactions [5]. This paper presents various important topics for discussion.

In their study of 16 prostate cancer patients undergoing initial ^177^Lu-PSMA RLT, Schumann et al. examined DNA damage in blood samples taken before and at seven time points (between 1 and 96 h) after radionuclide administration [6]. This study was the first to report the induction and persistence of double-strand break (DSB) DNA damage combined with internal dosimetry after therapy with ^177^Lu-PSMA. The minimal, maximal, and average total absorbed doses to the blood were 76, 164, and 109 ± 28 mGy, respectively. The average number of radiation-induced foci (RIF) per cell increased within the first 4 h after administration, followed by a decrease indicating DNA repair. The number of RIF during the first 2.6 h correlated linearly with the absorbed dose to the blood, which was in good agreement with previously published in-vitro data [7]. DNA DSBs were repaired effectively in many patients; however, RIF did not disappear completely in some patients, even 96 h after administration. Parameters of kidney function did not correlate with the absorbed dose rate or with the average number of RIF. The authors recognized the necessity to conduct further clinical research in a larger number of patients.

There are few data regarding the feasibility of rechallenge ^177^Lu-PSMA-617 RLT. Yordanova et al. performed a retrospective review of survival, response, and adverse events in patients who underwent rechallenge PSMA RLT [8], and found no cases of life-threatening (i.e., Common Toxicity Criteria [CTC] grade 4) adverse events after retreatment. However, 23% of patients experienced serious impairment of bone marrow function, one patient developed serious renal impairment, and 70% of patients developed irreversible adverse events (of which 90% were low-grade and 10% were grade 3 toxicity). The difference in overall survival between patients who underwent rechallenge PSMA-RLT compared with those who received only baseline PSMA RLT was significant: 25 vs. 9 months, respectively. The authors concluded that retreatment with PSMA-RLT appears to be safe and effective, resulting in benefits for most patients who had previously responded to PSMA-RLT [8]. The findings of this paper are important when considering the indications for PSMA-RLT.

## PSMA-targeted radioligand therapy with new beta-ray and alpha-ray emitting radioisotopes other than ^177^Lu-PSMA monotherapy

In Europe single-agent ^177^Lu-PSMA RLT has been studied in clinical trials, but there is also great interest in alpha-ray-emitting radioisotopes with significant therapeutic effects and easy outpatient treatment management, as well as beta-ray-emitting radioisotopes and with Auger electrons emitting radioisotopes with a short range [9]. PSMA RLT labeled with alpha-ray-emitting radioisotopes is being used clinically in some countries, and has been reported increasingly. In Japan, where government regulations are strict and obtaining some therapeutic radioisotopes produced by the nuclear reactor is difficult, the introduction of alpha-ray-emitting radioisotopes other than Radium-223 (^223^Ra) is still insufficient. Thus, there are no reports of the use of PSMA RLT with alpha-ray-emitting radioisotopes thus far. Although delayed in some countries, manufacturing alpha-ray-emitting radioisotopes by cyclotrons is possible, with several results emerging despite its being still in the research stage [10, 11].

The most advanced clinical PSMA RLT study of alpha-ray-emitting radioisotopes involves Actinium-225 (^225^Ac)-PSMA-617, although in a report of 17 patients with advanced prostate cancer treated with chemotherapy, more than 91% showed a decrease in PSA levels of more than 50%, with 41% saying that it had dropped below the measurement limit. Even in patients who discontinued it during the treatment course due to bone marrow suppression and renal dysfunction, relief of bone metastasis pain was observed [12, 13]. It should be noted that a rare case of prostate cancer brain metastasis with ^225^Ac-PSMA-617 has been reported, and in small multiple brain metastasis cases, this treatment may be a useful option to consider [14].

^177^Lu-PSMA RLT is known to have only around a 30% effect, whereas PSMA RLT with alpha-ray-emitting radioisotopes has produced surprising results. Treatment with ^225^Ac-PRLT has been reported to result in dry mouth [9]. In an attempt to reduce this side effect, a study combined tandem PSMA RLT with low-dose administration of ^225^Ac-PSMA after ^177^Lu-PSMA, with improved therapeutic effects and reduction in side effects observed in 20 cases [15].

A study calculated the absorption dose based on the distribution of Bismuth-213 (^213^Bi)-PSMA RLT on ^68^Ga-PSMA PET, which is expected to be another alpha-ray-emitting radioisotope. Unfortunately, although it had greater off-target perfusion radioactivity than ^225^Ac-PSMA RLT, its therapeutic window was shown to be narrower than that of ^225^Ac-PSMA RLT [16]. After theoretical dose calculation, ^213^Bi seems to be the choice after ^225^Ac according to their report. Given its physical half-life, 8 h to 10 days is considered desirable in practical clinical practice, while for ^225^Ac and Lead-212 (^212^Pb), 9.9 days and 10.6 h, respectively, are promising [17]. According to the study, the dynamics of ^212^Pb-PSMA can be confirmed based on ^203^Pb-PSMA, although slightly inferior to the computational ^225^Ac-PSMA RLT, ^212^Pb has the possibility of being available using the ^224^Ra generator.

Among the therapeutic beta-ray-emitting radioisotopes, ^177^Lu is at the core of PSMA RLT. However, it has been reported that Terbium-161 (^161^Tb) has better characteristics than ^177^Lu from theoretical dose calculation (*T*1/2 = 6.89 days; Eβav = 154 keV) [18]. According to the report, because of the effect of Auger electrons in addition to beta rays in ^161^Tb-PSMA-617, it is possible to deliver higher absorption doses to tumor cells than if ^177^Lu was used. Further, low-energy gamma rays suitable for SPECT (*Eγ* = 49 keV, *I* = 17.0%; *Eγ* = 75 keV, *I* = 10.2%) are also emitted. Therefore, imaging and dosimetry are also possible at the same time. In general, targeted radioligand therapy is effective for the treatment of small metastatic cells. Since the effect of ^161^ Tb is high and its range of radiation is short, it seems to be preferable as can be seen from the current situation where it is being replaced by ^177^Lu rather than Iodine-131 (^131^I) or yttrium-90 (^90^Y).

## Diagnostic performance of ^68^ Ga-PSMA PET

^68^Ga-PSMA is one of the promising PET tracers for detecting prostate cancer in various clinical scenarios. Recently, there are many articles published in patients with very low PSA to predict the diagnostic ability of ^68^Ga-PSMA PET. Farolfi et al. reported the diagnostic performance of ^68^Ga-PSMA PET/CT in 119 prostate cancer patients with biochemical recurrence retrospectively, whose PSA levels are the range 0.2–0.5 ng/ml at the time of PET. ^68^Ga-PSMA PET/CT was positive in 34.4%, and clinical physicians changed their treatment strategy in 30.2% [19]. Ceci et al. demonstrated the diagnostic usefulness of ^68^Ga-PSMA PET/CT prospectively in 332 patients with biological recurrence (PSA 0.2–2.0) [20]. The detection rate in this study was 53.6% and was different depending on the clinical stage of biological recurrence. These results reveal that ^68^Ga-PSMA PET/CT can detect very early recurrence in at least 30% of patients.

Magnetic resonance imaging (MRI) is one of the common imaging modalities to diagnose prostate cancer, and recently several studies compared the diagnostic performance of multi-parametric MRI (mpMRI) with ^68^ Ga-PSMA PET/CT. A retrospective study showed 43 patients with recurrent prostate cancer who underwent MRI with ^68^ Ga-PSMA PET/CT within 2 months. ^68^ Ga-PSMA PET/CT detected more pelvic lesions characteristic for recurrent prostate cancer (75 lesions) when compared to MRI (53 lesions) [21]. Another retrospective study revealed 58 patients who underwent MRI with ^68^ Ga-PSMA PET/CT followed by radical prostatectomy. ^68^ Ga-PSMA PET/CT correctly detected more foci (78%, AUC 0.817) than mpMRI (69%, AUC 0.729) [22]. ^68^ Ga-PSMA PET/CT may better reflect the histopathology of radical prostatectomy compared to mpMRI when considering multifocal and bilateral disease. The notable points of these articles are not only the high diagnostic performance of ^68^ Ga-PSMA PET/CT but also possibly include ^68^ Ga-PSMA PET/CT for the initial staging of recurrent prostate cancer in the clinical algorithms.

From the viewpoint of clinical impact, ^68^ Ga-PSMA PET/CT led to a change in management in 60% with recurrent prostate cancer [23]. The proportion of patients in whom systemic therapy was selected decreased from 60 to 34% based on the information provided by the ^68^ Ga-PSMA PET/CT scan. In another cohort of the early biochemical relapse setting following radical prostatectomy, ^68^ Ga-PSMA PET/CT led to a management change in 42.8% [24]. These articles suggested that some clinical impact of ^68^ Ga-PSMA may be seen in about half of prostate cancer patients. In summary, ^68^ Ga-PSMA is a tracer outgrowth that has the potential to significantly change treatment strategies for prostate cancer.

## ^68^Ga-PSMA PET/CT beyond prostate cancer

The role of ^68^ Ga-PSMA PET/CT in the staging of prostate cancer has been widely reported. As PSMA is also overexpressed in the neovasculature of other tumours [25], it has a great potential for the diagnosis of cancer beyond prostate cancer [26].

Raveenthiran et al. evaluated the value of ^68^ Ga-PSMA PET/CT in the investigation and management decisions of renal cell carcinoma. ^68^ Ga-PSMA-avid primary lesions were identified in 75% of cases, the majority of which were clear cell subtype. The ^68^ Ga-PSMA PET/CT findings altered the clinical management in 43.8% of the primary staging patients and in 40.9% of those undergoing restaging, and directly changed the management in 42.1% of patients following the identification of new sites of suspected metastases and the detection of synchronous primaries [27].

Metastasis to organs can also be identified by ^68^ Ga-PSMA PET/CT. Sathekge et al. reported highly positive ^68^ Ga-PSMA PET/CT in cerebral metastases and widespread skeletal metastases. Identified ^68^ Ga-PSMA-positive lesions can be treated using recently introduced ^177^Lu-PSMA-617 RLT and ^225^Ac-PSMA RLT. Sathekge et al. described a patient with brain metastasis from prostate cancer who was treated with ^225^Ac-PSMA ligand. The patient was treated with one cycle of ^225^Ac-PSMA-617 with activity of 8 MBq. After one cycle of ^225^Ac-PSMA-617 therapy, ^68^ Ga-PSMA PET/CT demonstrated marked functional and biochemical responses [14]. This case highlights the potential of ^225^Ac-PSMA-617 for treatment of brain metastases from metastatic castration-resistant prostate cancer with limited treatment options. Therefore, the potential of PSMA for identification of metastasis and cancer beyond prostate cancer appears to be substantial in terms of patient management and subsequent alpha and beta radiation therapy.

Pfob et al. reported parathyroid adenoma as an incidental finding on ^68^ Ga PSMA PET/CT [28]. It is important to understand the physiological uptake of PSMA ligand in PSMA-avid benign lesions to avoid misdiagnosis and the possible side effects of subsequent alpha and beta radiation therapy. As the number of studies reporting incidental findings on ^68^ Ga PSMA PET/CT has increased recently, we need to regularly update our understanding of the behavior ^68^ Ga PSMA PET/CT [26].

In surveying for organ-specific disease, the most precise imaging modality will greatly increase the diagnostic sensitivity. Morsing et al. reported the results of a hybrid PET/MRI study of major cancers [29]. They considered that PSMA PET can be most effectively employed in PET/MRI because of the superior image quality of MRI imaging of the pelvis compared with PET/CT, which will in turn improve accuracy in the assessment of disease extent and the discrimination of benign lesions from physiological uptake.

Understanding the potential of ^68^ Ga PSMA PET/CT as an emerging imaging modality will enable the further application of PSMA ligand.

## Conclusions

In this review article, we briefly overviewed the current status of PSMA RLT and PSMA PET. We hope that this topic will be a particular focus of attention for most ANM readers in Japan, and that our efforts will facilitate the early approval of PSMA RLT and PSMA PET by the Japanese government even if only slightly.
